# The Effect of Differential Growth Rates across Plants on Spectral Predictions of Physiological Parameters

**DOI:** 10.1371/journal.pone.0088930

**Published:** 2014-02-11

**Authors:** Tal Rapaport, Uri Hochberg, Shimon Rachmilevitch, Arnon Karnieli

**Affiliations:** 1 The Remote Sensing Laboratory, The Jacob Blaustein Institutes for Desert Research, Ben Gurion University of the Negev, Beer-Sheva, Israel; 2 The French Associates Institute for Agriculture and Biotechnology of Drylands, The Jacob Blaustein Institutes for Desert Research, Ben Gurion University of the Negev, Beer-Sheva, Israel; University of Vigo, Spain

## Abstract

Leaves of various ages and positions in a plant's canopy can present distinct physiological, morphological and anatomical characteristics, leading to complexities in selecting a single leaf for spectral representation of an entire plant. *A fortiori*, as growth rates between canopies differ, spectral-based comparisons across multiple plants – often based on leaves' position but not age – becomes an even more challenging mission. This study explores the effect of differential growth rates on the reflectance variability between leaves of different canopies, and its implication on physiological predictions made by widely-used spectral indices. Two distinct irrigation treatments were applied for one month, in order to trigger the formation of different growth rates between two groups of grapevines. Throughout the experiment, the plants were physiologically and morphologically monitored, while leaves from every part of their canopies were spectrally and histologically sampled. As the control vines were constantly developing new leaves, the water deficit plants were experiencing growth inhibition, resulting in leaves of different age at similar nodal position across the treatments. This modification of the age-position correlation was characterized by a near infrared reflectance difference between younger and older leaves, which was found to be exponentially correlated (R^2^ = 0.98) to the age-dependent area of intercellular air spaces within the spongy parenchyma. Overall, the foliage of the control plant became more spectrally variable, creating complications for intra- and inter-treatment leaf-based comparisons. Of the derived indices, the Structure-Insensitive Pigment Index (SIPI) was found indifferent to the age-position effect, allowing the treatments to be compared at any nodal position, while a Normalized Difference Vegetation Index (NDVI)-based stomatal conductance prediction was substantially affected by differential growth rates. As various biotic and abiotic factors may form distinctions in growth, future precision agriculture studies should consider its spectral effect on physiological predictions.

## Introduction

Over the last few decades, improvements in the spatial and spectral capabilities of remote sensors have significantly promoted the precise monitoring of crop growth and development, from the field scale to the satellite level [1]–[4]. These technological advantages can provide a great assistance to farmers, provided that the variability within a plant's canopy – originating from spatial and phenological differences [5] – is understood. Physiologically, leaves of various ages and positions in a single plant were previously documented to have distinct levels of water content [6]–[8], water use efficiency [9], [10], stomatal conductance [11], [12], nitrogen content and allocation [13]–[15], photosynthesis [16]–[19], chlorophyll content [19]–[21], assimilation rates [12], [22], [23], and cellular structures and processes [24]. These within-canopy variations highlight the difficulty of representing an entire plant and, more so, comparing between different plants using a single leaf – normally chosen on the basis of the ‘age’ and ‘position’ concepts, which many studies refer to as equals (e.g., [25]–[28]). Indeed, when plants grow at a uniform rate, a constant age-position correlation can be assumed across them [22], meaning that leaves of a similar position are plausibly of a similar age. However, differences in leaf longevity or in growth rates, caused by genotypic differences [29] and biotic or abiotic stresses [30], can significantly modify this correlation [22], [31]. This hypothesis was already tested four decades ago by Wilson and Cooper [32], who concluded that “Differential rates of leaf appearance among plants may also affect comparisons”, and is especially notable for deciduous and annual plants that regrow their canopy on a yearly-basis. Dwyer and Stewart [33] reaffirmed this conclusion by showing that maximal photosynthetic capacity in maize correlates with leaf age but not with position. Generally, it seems that leaves can gain mature characteristics during growth inhibition, regardless of their position [34]. The specific control mechanism for this phenomenon, which is likely genetically- and environmentally dependent, is yet to be elucidated.

From a spectral perspective, Shibata [35] was one of the first to address the age-dependent variance within a canopy, when he correlated optical absorbance with chlorophyll formation in *Phaseolus vulgaris* leaves of different ages. Since then, pigment concentrations have been related to visible and infrared differences between young and mature leaves of *Hedera helix* [36], *Carica papaya* [20], *Pinus taeda* [37], [38], and of many other plant species (e.g., [39]–[43]). Spectral variability was also linked to changes in age-dependent histological variables within the leaf, such as the thickness of its tissues [44]–[46] and the air cavity distribution in its parenchyma layers [47]–[49]. The latter feature has been mainly linked to near infrared (NIR) reflectance, as the difference between the refraction index of cell walls and that of intercellular air voids – spaces that generally spread with leaf maturity – is likely the cause for scattering in this portion of the electromagnetic spectrum [50]–[54]. To date, only few remote sensing studies seemed to emphasize the importance of leaf age variability within a canopy in the context of spectral comparisons between plants. The most relevant and recent exception is the work of Liu *et al.* [41], who argued that “Few reports had simultaneously analyzed the relationship among the spectral reflectance indices, pigment concentrations, different plant species and developmental stages”. Their conclusion that “Some indices show greater promise as estimators of pigment concentrations than others” supports the ideas of Stone *et al.* [21], who stated that “Leaf-age composition of crowns needs to be taken into account when applying reflectance-based indices”. Another related example is the work of Isaacson *et al.* [55], who tried to normalize the phenological effect for common spectral indices using Landsat and MODIS data.

The current study was set to explore the effect of differential growth rates on the reflectance variability of leaves across different plants, and its impact on spectral-based physiological predictions. Specifically, the objectives were (1) to model the variance between individual leaves of canopies in the face of differential growth rates; (2) to relate this spectral variance among leaves of different age and position to the anatomical variance that causes it; and (3) to test the susceptibility of several widely-used reflectance indices to this spectral-anatomical effect, and their ability to predict physiological parameters in light of differential growth rates. To accomplish these objectives, two groups of ten grapevines were subjected to distinct water treatments, so that different growth rates could be established between them. During their development, the plants were physiologically and morphologically monitored in order to associate their water status to growth regulation. Additionally, leaves from every part of their canopies were spectrally and histologically sampled, so that reflectance measurements can be correlated with leaves' strata thickness and air cavity distribution. It was hypothesized that leaves of the same nodal position across the two irrigation groups could be gradually distinguished by their NIR reflectance values, due to anatomical differences, consequently limiting the ability of some spectral indices to physiologically compare between the stress treatments.

## Methodology

### Plant Materials and Growth Conditions

Twenty Cabernet Sauvignon one-year-old plants (*Vitis vinifera* L. cv.), grafted on Richter-110 rootstocks, were grown under controlled conditions in 10 liter pots filled with 9 liters of ‘RAM8’ media (Tuff Merom Golan, Merom Golan, Israel). The soil was enriched with a controlled-release fertilizer, and fresh water was applied on a daily basis through drip-irrigation in a rate of 2 L h^−1^. Day and night temperatures within the greenhouse were maintained between 28±1.5°C and 18±1.5°C, respectively. Plants were pruned to allow the growth of a single branch, while flower buds were removed upon emergence to sustain the vines in a vegetative state.

### Experimental Design

Until the beginning of the experiment, all twenty plants were irrigated daily to field capacity. The experiment began once the plants had reached a size of 12–16 leaves and lasted for 30 days. While ten vines were kept on the same irrigation regime and maintained as controls (C), the other ten plants were subjected to a steady level of water deficit (WD). The stress treatment was applied according to the suggestions of Pou *et al.* [56] and is briefly described as follows. Irrigation was halted from day 1 until a volumetric water content (VWC) range of 5–10% was achieved in the pots. The plants were then kept at this stress level by daily replenishments of the amounts of water consumed, which were determined by weighing the pots every evening and subtracting the results from their corresponding target weights. All pots were arranged in the greenhouse according to a randomized complete block design, and were put sufficiently far from one another and from the greenhouse edges to minimize shading effects.

Differences in the stress level of the vine groups were physiologically assessed by measuring selected gas exchange parameters. These measurements were taken on the 1^st^ day of experiment (DOE) before the stress was applied, on the 7^th^ DOE in order to allow the WD pots to reach the desired VWC, and on the 29^th^ DOE that was the last measuring day. Changes in vegetative development were evaluated by counting the number of nodes and measuring foliage area on the 1^st^, 15^th^, and 29^th^ DOE. In order to keep track of leaves' age, they were marked upon reaching a size of 1 cm^2^. Spectral measurements of the vines' leaves were taken on the 1^st^, 7^th^, 15^th^, and 29^th^ DOE. Tissue samples were collected on the 29^th^ day for complementary histological measurements. All types of measurements (Table 1) were conducted between 10:00–13:00.

**Table 1:** Summary of all measurement types that were taken during the experiment.

	Measurement type			
DOE	Gas exchange	Vegetative development	Spectral	Histological
1	X	X	X	
7	X		X	
15		X	X	
29	X	X	X	X

Measurements were taken on days of experiment (DOE) that are signified by X.

### Physiological and Morphological Measurements

Stomatal conductance (g_s_) and net CO_2_ assimilation rates (A_N_) of the youngest, fully-matured leaf of each plant were collected using a portable Li-6400 Infrared Gas Analyzer (Li-Cor Biosciences Inc., NE, USA). The open photosynthesis system was equipped with an external carbon dioxide source, in order to maintain the leaf chamber at a concentration of 400 µmol mol^−1^. Temperature and relative humidity range were 28°C and 30–55%, respectively, and the photosynthetic active radiation (PAR) rate was 1000 µmol photons m^−2^ s^−1^. Gas exchange measurements were taken inside the greenhouse.

Foliage area was determined using a regression model that correlated the surface dimensions of a leaf with its actual area. After logging the width and length of 30 leaves, their pictures were then taken against a white background. The images were then made binary using The GIMP 2.6.11 software (http://www.gimp.org), so that the real areas could be derived from the sum of all relevant pixels. Eventually, a least squares linear regression model was found to best predict the actual leaf area from width and length (Image area  = 1.72× Squared area +12.1; R^2^ = 0.95; *p*<0.01).

### Spectral Measurements

At every time-point, an ASD Pro-FR Field Spectroradiometer (Analytical Spectral Devices Inc., CO, USA), equipped with an 1800-12S Integrating Sphere (Li-Cor Biosciences Inc., NE, USA), was used to log the signature of the oldest leaf in every fourth node of each vine, starting from the terminal bud. The system was set to measure mean radiance units (each measurement was averaged out of 20 scans) in the 350–2500 nm range, at 1 nm spectral resolution. Normalized values of leaf reflectance (ρ) were derived from complementary energy flux measurements of white-reference standards (BaSO_4_ tablets) and dark current, as suggested in the Integrating Sphere's manual. The reflectance data was used to exert several narrow-band, water stress-related indices: Normalized Difference Vegetation Index (NDVI; [57]; (ρ800 nm−ρ670 nm)/(ρ800 nm +ρ670 nm)), Water Index (WI; [58]; (ρ900 nm/ρ970 nm)), Normalized Difference Infrared Index (NDII; [59]; (ρ820 nm−ρ1650 nm)/(ρ820 nm +ρ1650 nm)), Moisture Stress Index (MSI; [59]; (ρ1600 nm/ρ820 nm)) and Structure Insensitive Pigment Index (SIPI; [60]; (ρ800 nm−ρ445 nm)/(ρ800 nm−ρ680 nm)).

### Anatomical Measurements

Samples were collected from every 4^th^ leaf in a plant – over five repetitions per treatment – starting from the terminal bud and ending at the twentieth leaf from the top. Cross sections (transversal cuts) were processed according to the protocol of Rewald *et al.* [61], which was slightly modified. Photomicrographs of the cross sections were then taken with an Axio Imager A1 Light Microscope (Carl Zeiss Microscopy Inc., TH, Germany) and ×100 magnification images were produced using its AxioVision 4.6.3 software. The GIMP software was then used for measuring the thickness of all leaf tissues, i.e., both cuticle, epidermal, and parenchyma layers, and in obtaining the percentage of intercellular air voids' area from the entire adaxial and abaxial mesophyll surface. The latter analysis was performed by making each section image binary, summing all void pixels in each mesophyll layer, and normalizing each area result to the total cell region of the respective parenchyma.

### Statistical Analyses

All statistical calculations were performed using the SigmaPlot 12.0 software (Systat Software Inc., IL, USA). Differences between mean values were assessed using one-way Analysis of Variation (ANOVA) test, which was followed by Tukey's Honest Significance Difference (HSD) post-hoc test whenever necessary. Prior to those tests, assumptions of residuals' normality and homoscedasticity were checked and met using Shapiro-Wilk and Bartlett's tests, respectively. Least squares regressions were used in order to describe the continuous relationship between various variables. Results of all comparison tests and correlation analyses were considered significant at *p*<0.05.

## Results

### Physiological and Morphological Analyses

**Figure 1:**
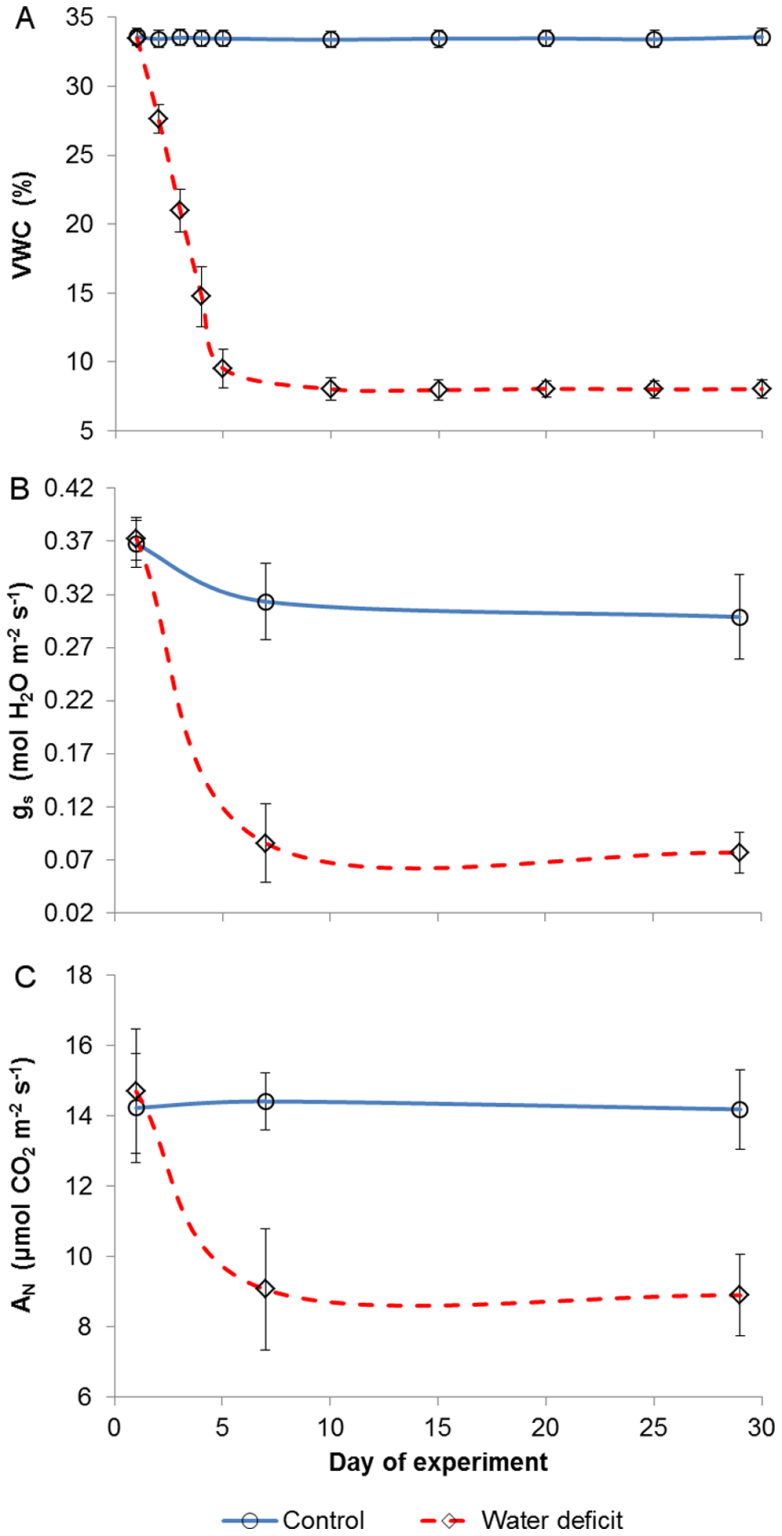
Volumetric water content (A; VWC), stomatal conductance (B; g_s_) and net assimilation rate (C; A_N_) throughout the experiment. Excluding day 1, values of the two groups were significantly different for every measuring date. Vertical bars represent means ± standard deviations (n = 10).

The water range for the WD pots was achieved between the 5^th^ and 6^th^ DOE, and was maintained at an average of 8% until the end of the experiment (Figure 1A). This water level was significantly different from that of the control pots, which were continuously refilled to a VWC of 33% by day 30. Significant physiological distinctions between the irrigation groups were noticed on the 7^th^ DOE, as the stomatal conductance (Figure 1B) and net assimilation rates (Figure 1C) of the WD vines were lower than those of the control vines by 0.23 mol H_2_O m^−2^ s^−1^ (a 75% reduction) and 5.35 µmol CO_2_ m^−2^ s^−1^ (a 37% reduction), respectively. Those gas exchange differences remained similar until the end of the experiment.

**Figure 2:**
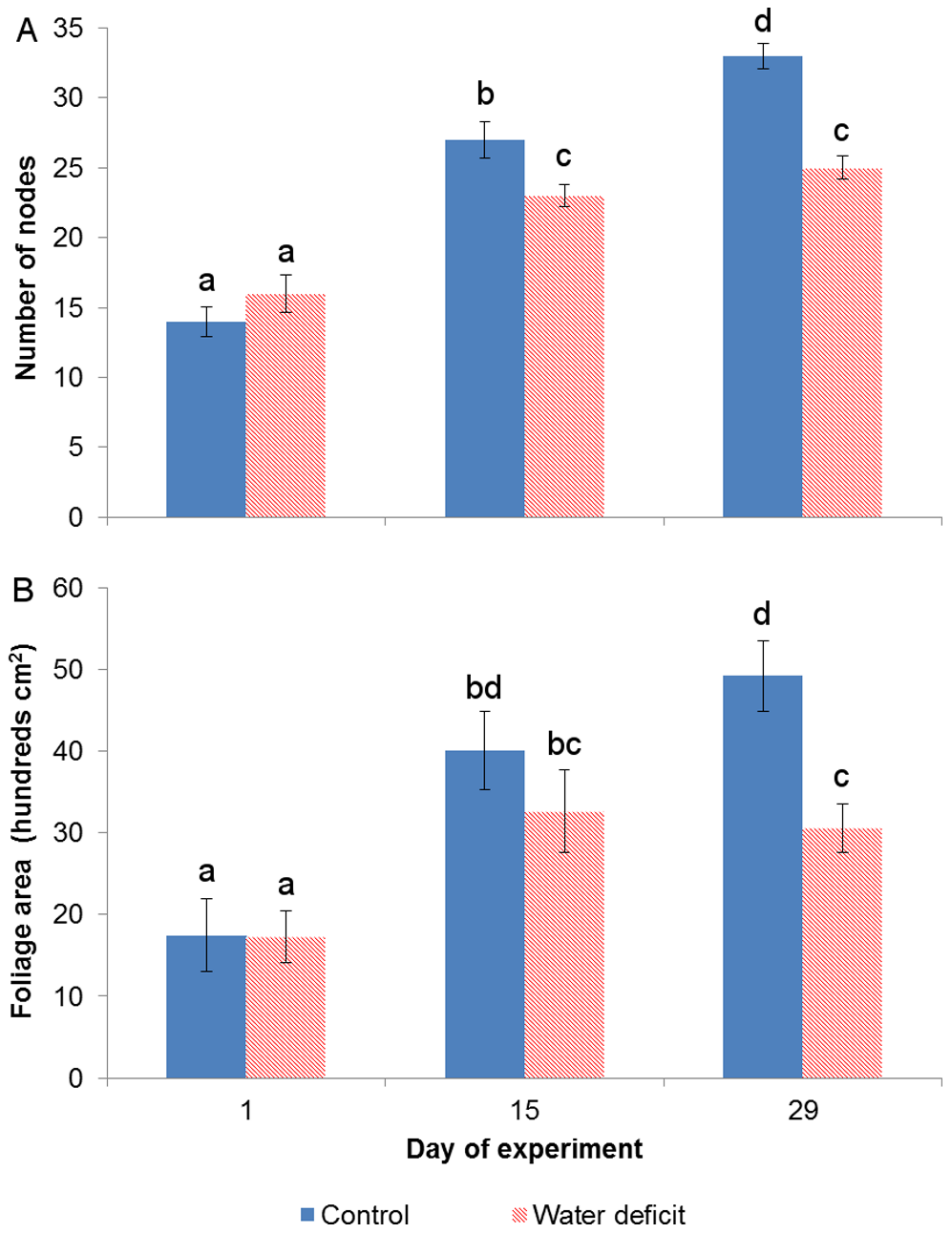
Number of nodes (A) and foliage area (B) throughout the experiment. Vertical bars represent means ± standard deviations (n = 10). Lower-case letters indicate significance of differences.

While the average control plant began the trial 2 nodes shorter than the average WD plant, the former concluded the experiment with 11 nodes more than the latter (Figure 2A). In terms of total leaf area, although both treatments were found similar at day 1 with 1730 cm^2^, the WD vines' foliage was 1860 cm^2^ smaller (a 40% reduction) than that of the control vines by the 29^th^ DOE (Figure 2B). Growth inhibition was apparent in the water deficit treatment from the 3^rd^ week onward, as its morphological values of the 15^th^ DOE were insignificantly different from those of day 29.

### Spectral Analyses

**Figure 3:**
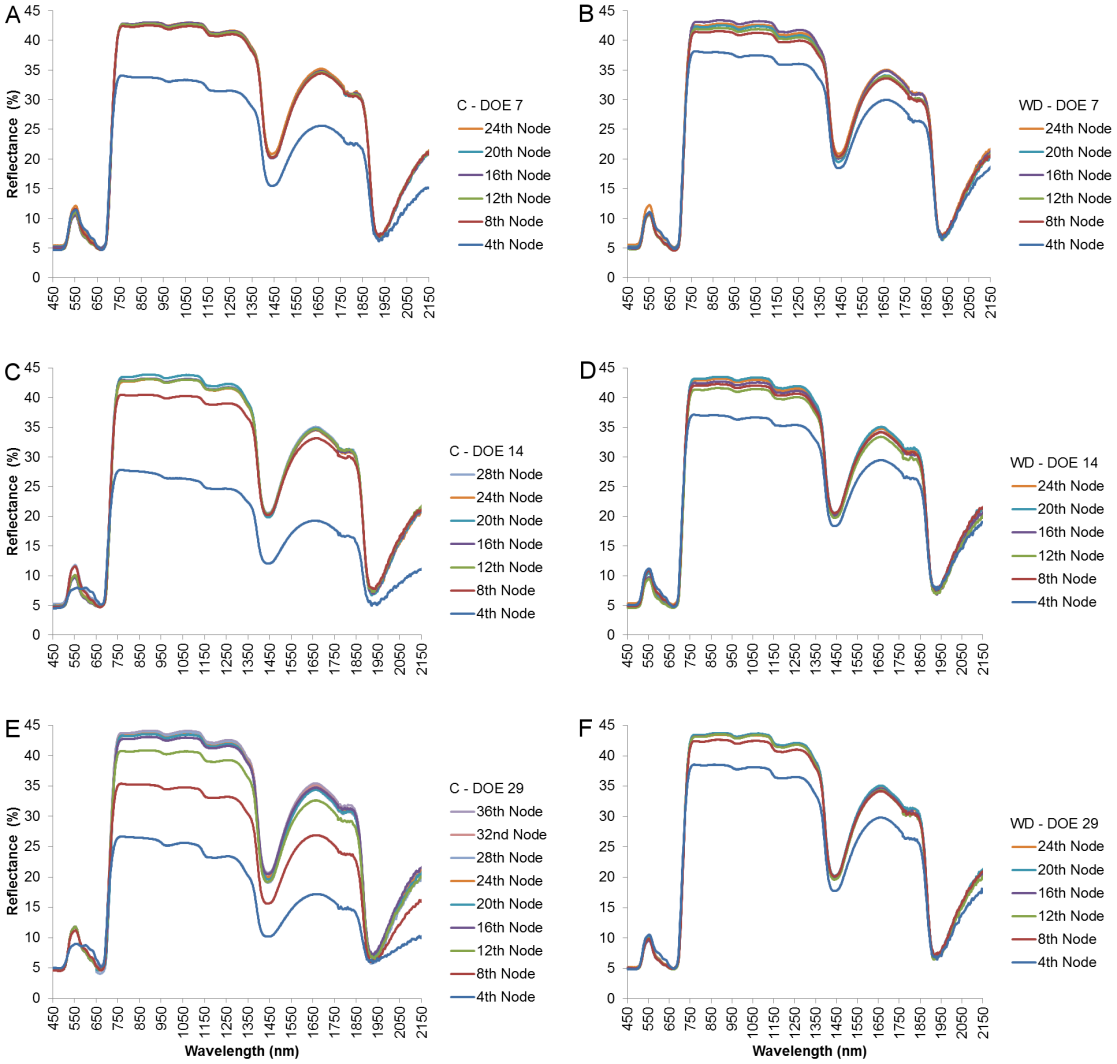
Reflectance of leaves from different nodal positions as a function of wavelength throughout the experiment. The reflectance signatures presented are of the 7^th^, 14^th^, and 29^th^ days of measuring for the control (A, C, and E, respectively) and water deficit (B, D, and F, respectively) groups. Each reflectance signature was averaged out of 10 leaf samples.

**Figure 4:**
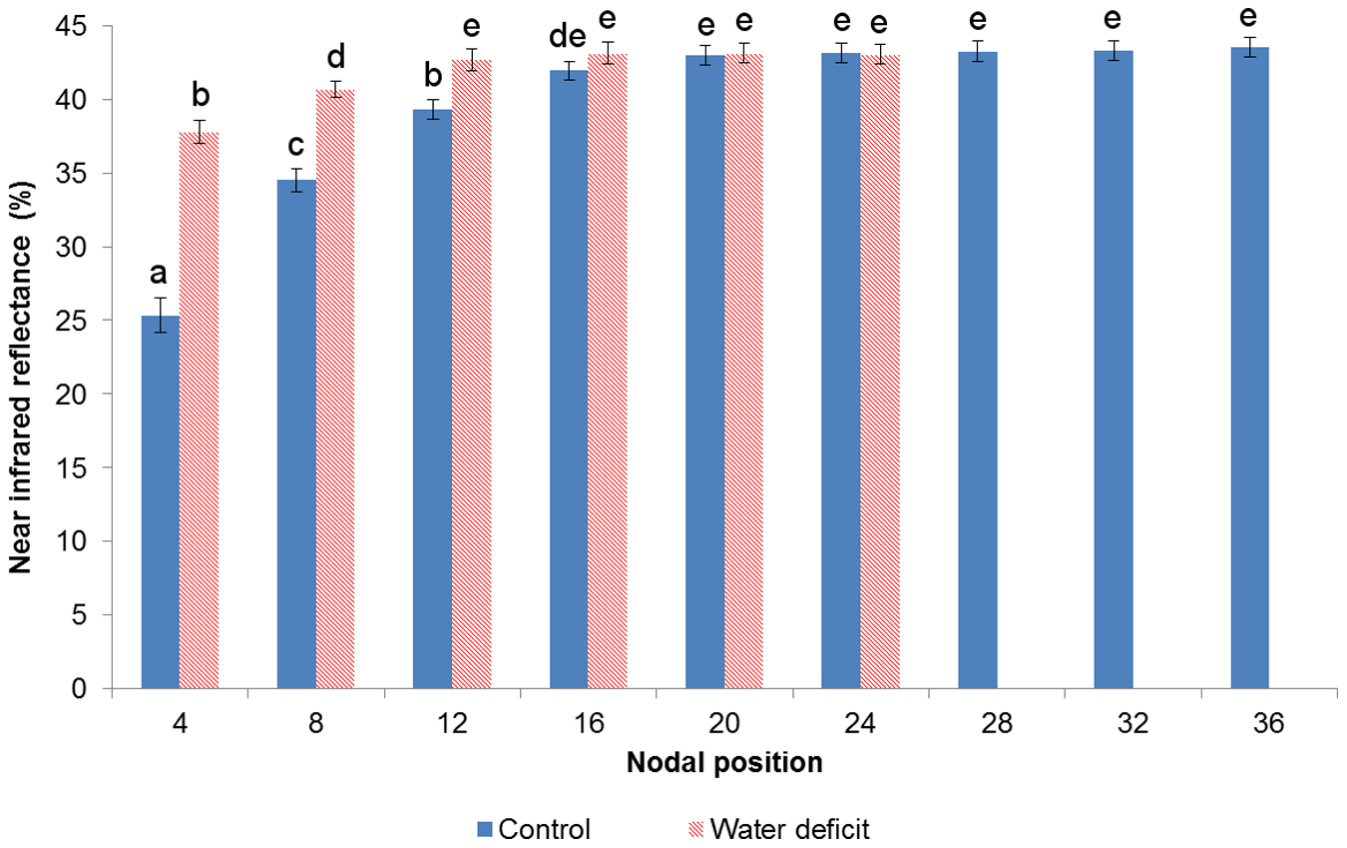
Near infrared reflectance of leaves as a function of their nodal position at day 29. Significant differences in the means of near infrared reflectance (750–1250 nm) were found between control and stressed leaves of the same nodal position down until the 16th node. The reflectance values in the upper 15 and 11 nodes of the control and water deficit groups, respectively, were significantly different from those in the rest of the nodes, and thus were not representative for most of the plant. Vertical bars represent means ± standard deviations (n = 10). Lower-case letters indicate significance of differences.

By the end of the first week of the experiment, the reflectance signatures of leaves in the control vine (Figure 3A) were still similar to those of the WD plant (Figure 3B). During the second week, from day 7 to day 14, the 4^th^ leaf of the control treatment has moved to the 8^th^ nodal position, presenting a 7% NIR reflectance increase (measured by taking the average of 750–1250 nm; Figure 3C). On the other hand, the 4^th^ leaf of the WD vine maintained its position, displaying only a minor reflectance transition (Figure 3D). As the foliage of the control plant continued to expand by day 29 (Figure 2), the NIR values of its younger leaves, down until the 12^th^ node, were significantly lower than those of its older leaves (downward from the 16^th^ node; Figure 3E). In the WD treatment, however, no new leaves emerged, while the existing ones maintained their position and their reflectance spectrum (Figure 3F). Eventually, by the end of the experiment, significant differences in NIR reflectance were found between control and stressed leaves of the same position down until the 16^th^ node. At that time, the average control and WD plants could have been spectrally-represented solely by leaves in their 24 and 16 lower nodes, respectively, as their reflectance values were similar and the majority in each canopy (Figure 4).

The effect of growth rates on selected spectral indices was assessed on the 29^th^ DOE (Table 2). In the water deficit treatment, characterized by low growth rates, most indices presented uniformity in values for all leaves measured – regardless of their position. This was a direct result of the similar reflectance signatures of leaves in the majority of nodes. In contrast, most control indices displayed value inconsistencies at higher leaf positions (4^th^-12^th^ nodes). The greatest variation was observed in the NDVI and WI profiles, wherein leaves at the top 12 nodes (between 41–55% of the foliage) showed significantly different values as compared with leaves in the rest of the canopy. The least susceptible indices, second only to the indifferent SIPI, were the NDII and MSI that were insensitive to value changes at the bottom 32 nodes (between 59–73% of the foliage).

**Table 2:** Spectral indices distribution along the control (C) and water deficit (WD) canopies at day 29.

			Spectral indices				
	Node	% foliage	NDVI	WI	NDII	MSI	SIPI
C	4	27.3±4.0	0.651±0.034^A^	1.043±0.009^A^	0.213±0.034^A^	0.621±0.063^A^	**1.013**±**0.010^A^**
	8	41.5±4.9	0.738±0.016^B^	1.026±0.002^B^	**0.131**±**0.017^B^**	**0.733**±**0.015^B^**	1.007±0.007^A^
	12	54.7±4.6	**0.789**±**0.012^C^**	**1.016**±**0.002^C^**	0.114±0.007^B^	0.768±0.023^B^	1.004±0.004^A^
	16	65.7±3.5	0.797±0.016^D^	1.014±0.001^C^	0.113±0.006^B^	0.769±0.015^B^	1.000±0.005^A^
	20	74.5±2.7	0.799±0.011^D^	1.015±0.001^C^	0.113±0.005^B^	0.759±0.013^B^	1.004±0.006^A^
	24	83.1±2.3	0.801±0.012^D^	1.015±0.002^C^	0.114±0.004^B^	0.761±0.012^B^	1.005±0.004^A^
	28	92.6±1.6	0.800±0.009^D^	1.015±0.001^C^	0.114±0.003^B^	0.760±0.015^B^	0.999±0.004^A^
	32	97.5±1.5	0.798±0.009^D^	1.016±0.002^C^	0.113±0.004^B^	0.772±0.023^B^	1.003±0.004^A^
	36		0.801±0.008^D^	1.014±0.002^C^	0.114±0.007^B^	0.774±0.023^B^	1.002±0.003^A^
WD	4	31.8±2.3	0.752±0.012^A^	**1.015**±**0.004^A^**	**0.113**±**0.003^A^**	**0.748**±**0.030^A^**	**1.005**±**0.006^A^**
	8	43.8±1.8	**0.778**±**0.007^B^**	1.012±0.002^A^	0.110±0.003^A^	0.770±0.010^A^	1.000±0.005^A^
	12	56.7±1.7	0.780±0.011^B^	1.010±0.001^A^	0.109±0.002^A^	0.766±0.012^A^	0.993±0.004^A^
	16	70.8±1.9	0.783±0.008^B^	1.012±0.003^A^	0.108±0.004^A^	0.767±0.008^A^	0.993±0.006^A^
	20	84.1±1.9	0.781±0.009^B^	1.011±0.002^A^	0.111±0.004^A^	0.772±0.013^A^	0.995±0.003^A^
	24		0.780±0.009^B^	1.011±0.003^A^	0.109±0.003^A^	0.780±0.017^A^	0.994±0.004^A^

% foliage represents the cumulative foliage area measured by each nodal position, and includes also leaves of appropriate size from lateral shoots. Bolded values represent the uppermost node in the canopy from which all lower nodes exhibit similar values. Data represents means ± standard deviations (n = 10). Upper-case letters indicate significance of differences within each treatment.

**Figure 5:**
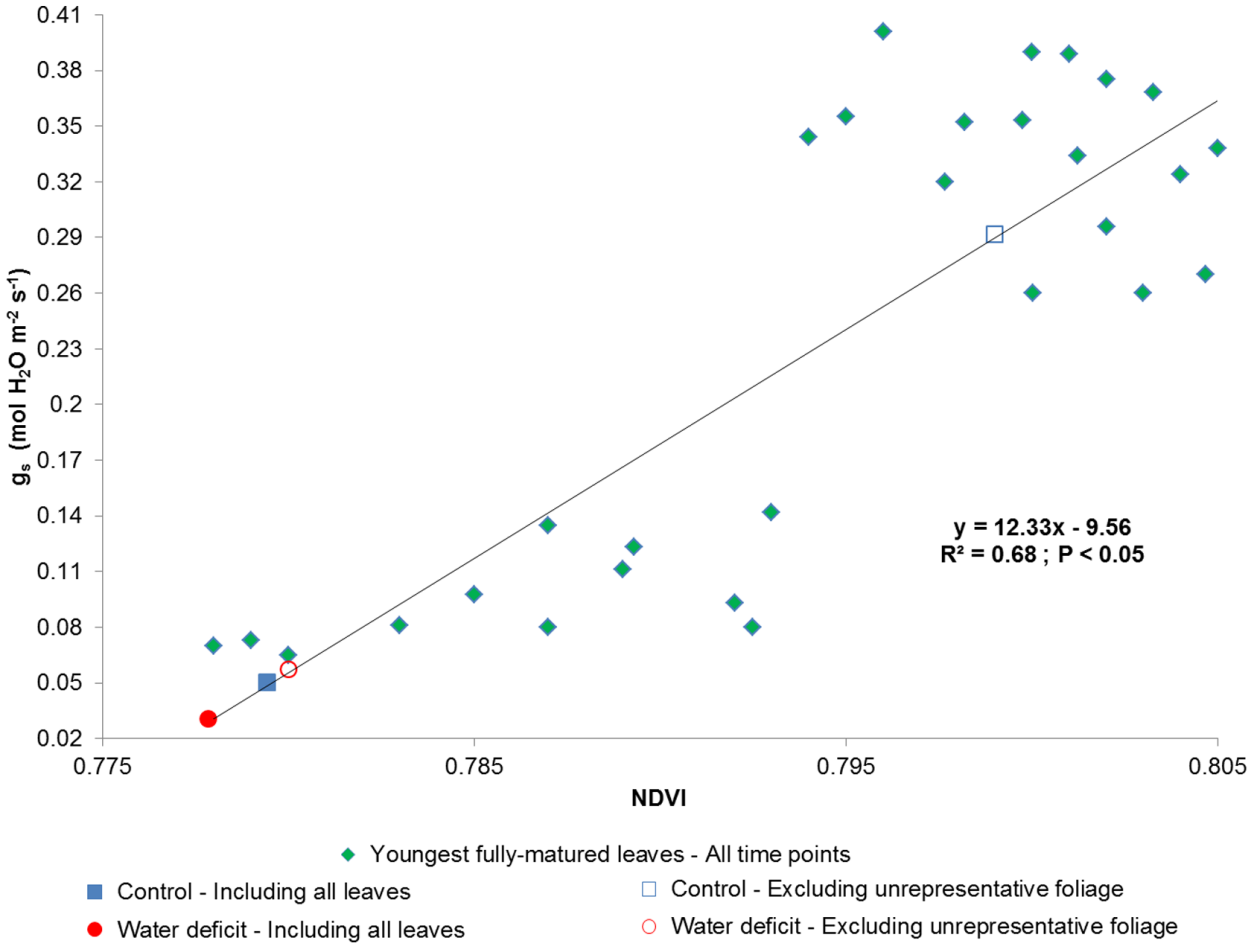
Stomatal conductance (g_s_) as a function of the Normalized Difference Vegetation Index (NDVI) throughout the experiment. The diamonds represent values of the youngest, fully-matured leaves of both treatments from all time points, and were used to create a linear regression. The filled square and circle are the 29^th^ day's mean values of leaves from the whole control and water deficit canopies, respectively. The unfilled square and circle are the 29^th^ day's mean values from only the representative portion of the control and water deficit canopies (Table 2), respectively.

To assess the extent of possible deviations caused by spectral variability, the NDVI values of the 29^th^ DOE (Table 2) were used. Throughout the experiment, the index values of twenty plants – calculated from the same youngest, fully-matured leaves that were used in the gas exchange measurements – were regressed against their stomatal conductance values, resulting in a statistically significant linear correlation (R^2^ = 0.68; *p*<0.05; Figure 5). For each treatment of the last measuring day, the regression was then used to compare the average g_s_ value of all measured leaves in the canopy to the average value predicted by only the leaves in the representative portion of the foliage (according to the NDVI value distribution shown in Table 2). Regarding the control vine, it was found that the exclusion of the 8 uppermost nodal positions, which were about 41% of the total foliage area, has increased the g_s_ rate from 0.050 to 0.292 mol H_2_O m^−2^ s^−1^. A smaller increase in stomatal conductance, from 0.031 to 0.057 mol H_2_O m^−2^ s^−1^, was observed when the 4 uppermost nodal positions were discarded from the WD vine, because – in comparison to the control canopy – the excluded leaves were only about 32% of the total foliage area and their NDVI values were of closer resemblance to those of older leaves in the canopy.

### Anatomical Analyses

**Figure 6:**
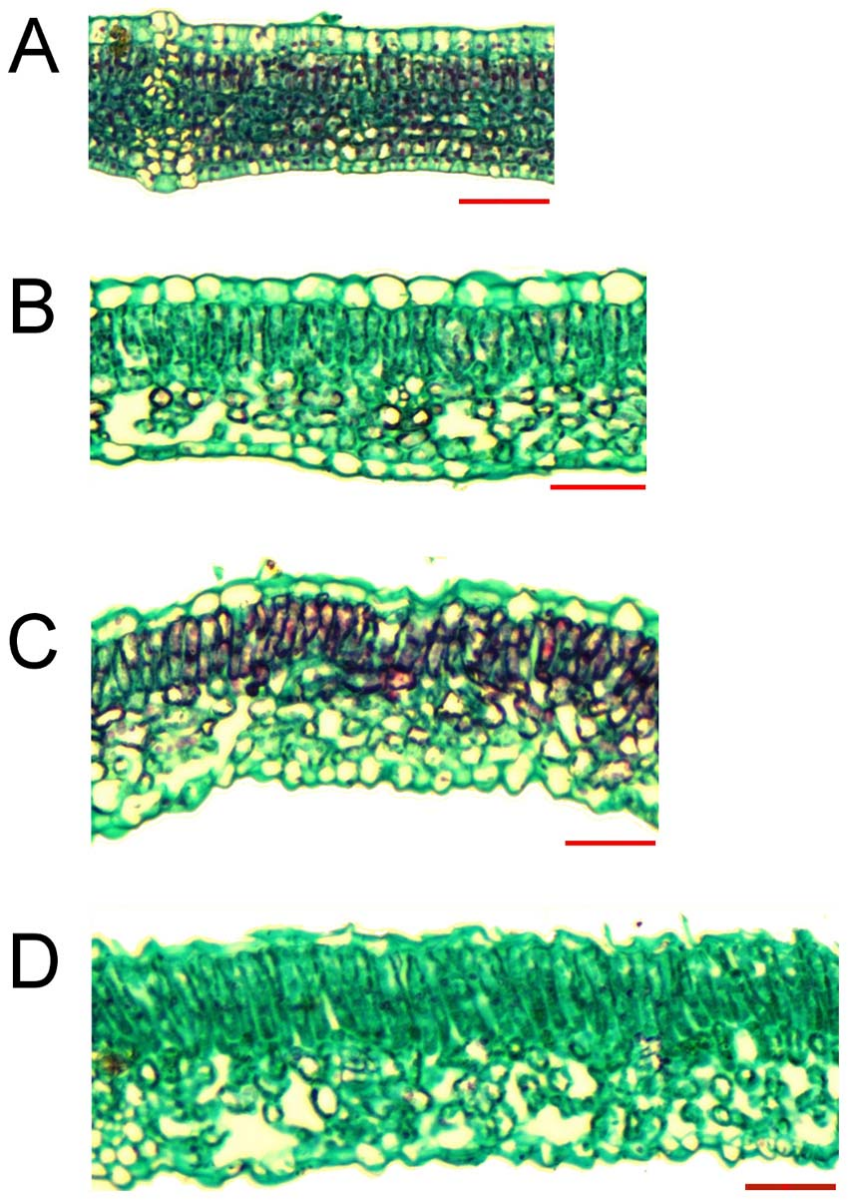
Leaf cross-sections at day 29. The scale bars of both control and water deficit's 4^th^ leaves (A and B, respectively) and 16^th^ leaves (C and D, respectively) equal 50 µm.

**Figure 7:**
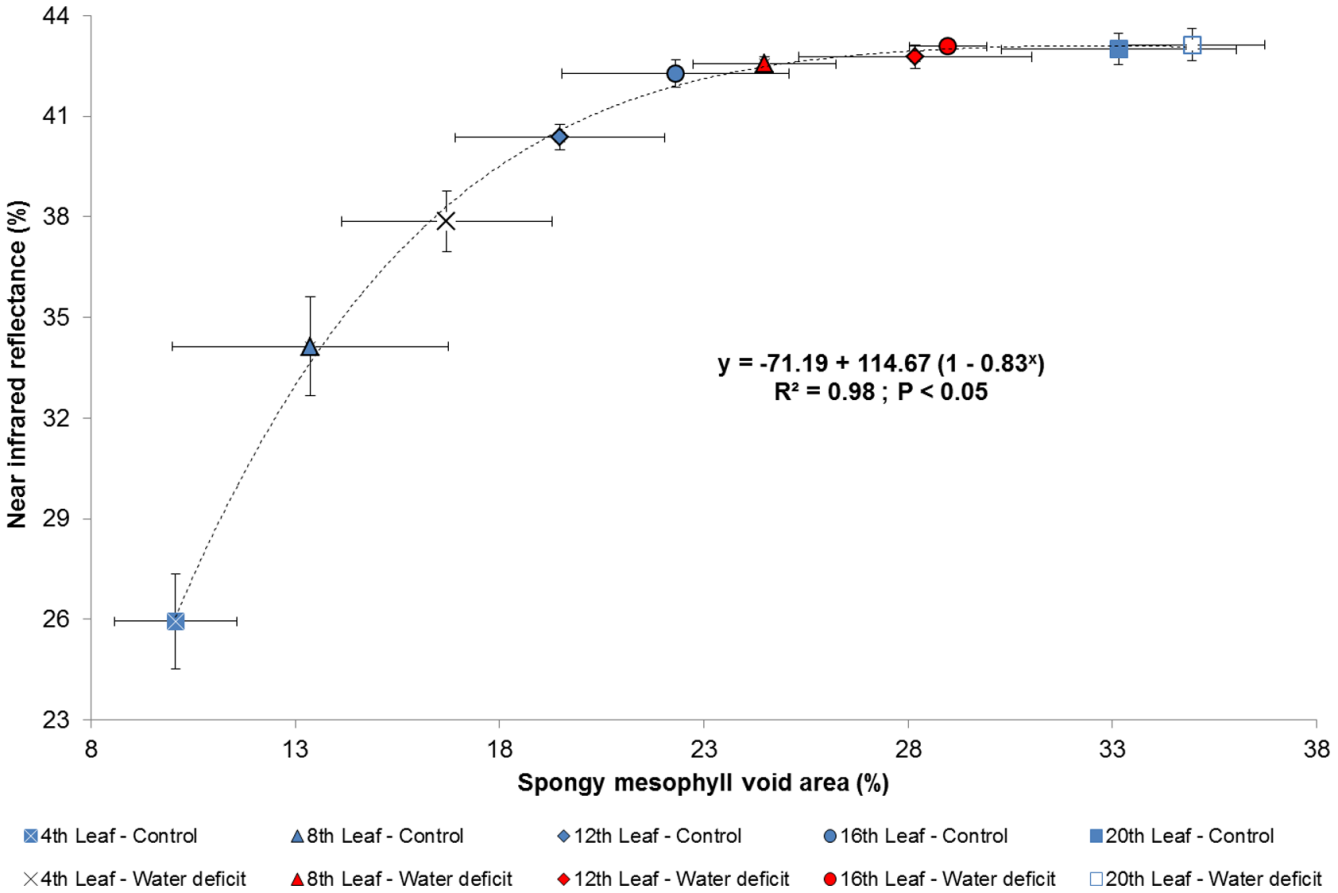
Near infrared reflectance of leaves as a function of their spongy mesophyll void area at day 29. Vertical and horizontal bars represent means ± standard deviations (n = 10 for the near infrared reflectance; n = 5 for the spongy mesophyll void area).

Thickness measurements of all leaf strata, including both cuticle, epidermal, and mesophyll layers, revealed insignificant differences between most nodes in both treatments (Table 3). Leaf position along the vine branches was also found to poorly affect the intercellular void areas within the palisade parenchyma (Table 4), as little evidence of air cavity expansion was found between young leaves of both the control and water stress treatments (Figures 6A and 6B, respectively) and their older counterparts (Figures 6C and 6D, respectively). However, leaf maturation in both treatments did result in a prominent visual increase in air cavities' area within the spongy mesophyll, backed up by significant differences between most nodal positions (Table 4). A statistically significant, strong exponential correlation (R^2^ = 0.98; *p*<0.05) was also found between the area of air spaces within the abaxial parenchyma of all leaves and NIR reflectance (Figure 7).

**Table 3:** Thickness of strata in leaves along the control (C) and water deficit (WD) canopies at day 29.

		Thickness (µm)						
	Node	UC	UE	CM	SM	LE	LC	WL
C	4	1.74±0.24^A^	9.73±2.98^A^	35.06±4.18^A^	38.03±3.02^A^	9.39±1.43^ABC^	1.44±0.52^A^	95.39±6.15^A^
	8	2.06±0.65^A^	10.49±2.19^A^	33.55±6.55^A^	42.17±4.86^AB^	9.83±0.69^BC^	1.70±0.15^A^	99.80±8.50^A^
	12	1.76±0.17^A^	9.08±2.39^A^	33.26±1.74^A^	42.02±6.57^AB^	10.38±1.75^ABCD^	1.45±0.23^A^	97.95±7.42^A^
	16	1.46±0.58^A^	8.48±1.92^A^	38.12±5.41^AB^	41.83±5.32^AB^	8.63±0.97^AB^	1.61±0.30^A^	100.13±7.91^AB^
	20	1.98±0.26^A^	8.97±1.39^A^	38.87±3.44^AB^	44.05±6.81^AB^	9.11±0.48^AB^	1.92±0.18^AB^	104.90±7.78^ABC^
WD	4	2.03±0.18^A^	11.39±1.22^A^	36.28±2.61^A^	42.40±1.88^A^	7.65±1.25^A^	1.88±0.39^ABC^	101.63±3.69^A^
	8	2.25±0.29^A^	11.61±1.72^A^	44.59±3.57^B^	43.46±1.55^AB^	12.27±2.25^CD^	2.50±0.27^C^	116.68±4.83^CD^
	12	1.94±0.17^A^	10.22±1.42^A^	42.25±1.09^B^	44.26±4.91^AB^	10.42±1.91^ABCD^	2.28±0.47^ABC^	111.37±5.59^BCD^
	16	2.32±0.29^A^	11.15±0.89^A^	43.47±0.97^B^	47.63±1.75^B^	11.82±0.46^D^	1.72±0.24^A^	118.11±2.27^D^
	20	1.84±0.54^A^	9.74±2.60^A^	45.18±2.45^B^	44.78±3.05^AB^	8.74±1.96^ABC^	2.24±0.22^BC^	112.52±5.12^BCD^

UC =  Upper cuticle; UE =  Upper epidermis; CM =  Column mesophyll; SM =  Spongy mesophyll; LE =  Lower epidermis; LC =  Lower cuticle; WL =  Whole leaf. Data represents means ± standard deviations (n = 5). Upper-case letters indicate significance of differences.

**Table 4:** Percentage of mesophyll void areas within leaves along the control (C) and water deficit (WD) canopies at day 29.

		Intercellular void area (%)	
	Node	CM	SM
C	4	2.10±1.21^A^	10.07±1.50^A^
	8	3.35±2.61^AB^	13.37±3.38^AB^
	12	5.29±2.87^AB^	19.48±2.27^C^
	16	5.82±3.21^AB^	22.31±2.78^CD^
	20	5.84±3.37^AB^	33.15±2.88^FG^
WD	4	6.16±3.36^AB^	16.71±2.58^BC^
	8	6.40±2.12^B^	24.49±1.74^DE^
	12	7.01±3.28^B^	28.18±2.85^EF^
	16	6.45±1.89^B^	28.97±0.95^E^
	20	7.22±3.22^B^	34.94±1.77^G^

CM =  Column mesophyll; SM =  Spongy mesophyll. Data represents means ± standard deviations (n = 5). Upper-case letters indicate significance of differences.

## Discussion

In order to investigate the spectral effects of differential growth rates on physiological predictions, Cabernet Sauvignon grapevines were subjected to two distinct irrigation regimes. Throughout the experiment, the stress level that was applied to the water deficit (WD) treatment group (Figure 1) can be regarded as moderate, and its occurrence under field conditions is not rare [62]–[64]. The observed, consequent down-regulation of stomatal conductance (g_s_) in the WD vines was likely the cause for their steeply-reduced CO_2_ assimilation rates (A_N_; Figure 1) [17], [65]–[68]. The A_N_ restrictions, in turn, were presumably responsible for the inhibition of vegetative growth in the water stressed group (Figures 2) [69], [70]. Since the expansion of the control vines' foliage was not limited by water shortage, a distinct correlation between leaves' age and position was gradually forming across the treatment groups. From a spectral perspective, the breaking of the age-position relation was mainly expressed through a growing difference in the NIR signatures of parallel leaves of a higher position (Figure 3). Namely, while a leaf of the WD group maintained its position along the branch but still continued to age and increase in reflectivity, its control counterpart was being constantly replaced by a younger leaf with significantly lower NIR reflectance values. In this regard, it should be noted that a differential effect of the water stress factor itself on the structure of young and mature leaves in the same plant cannot be ruled out [5], [34], [41], [71]. However, the results of previous studies, in which a similar spectral pattern of leaf maturation was shown in light of other stressing agents and conditions (e.g., [5], [20], [47], [52], [72], [73]), seem to support our proposed mechanism and suggest that the age variability mainly influenced the differences in reflectance. Therefore, it is argued that the canopy of the fully-growing plant became, over time, younger and more spectrally-variable than that of the stressed plant (Figure 4), creating complications for the selection of representative leaves for intra- and inter-treatment comparisons.

The aging of grapevine leaves is known to be accompanied by an increase in their mesophyll layers' intercellular air spaces [74]–[76]. As was previously suggested for various *Vitis vinifera* cultivars (e.g., [8], [77]), the dependence of NIR reflectance on leaf maturity is likely a derivative of compaction changes within the adaxial and abaxial parenchyma, as scattering in this spectrum generally increases with the increase in air void distribution in both mesophyll layers. In contrast to earlier studies that correlated NIR reflectance to the number of air voids in both mesophyll layers, to the total area of cell wall-air cavity refraction zones, or to the thickness of the leaves' strata (e.g., [39], [45]–[48], [53]), the results of this study indicate that NIR reflectance in a Cabernet Sauvignon leaf is mainly a function of the percentage of air spaces' area from the spongy mesophyll's area (Figures 6 & 7 and Table 4) and not of the thickness of any strata (Table 3). These findings are in agreement with those of Gausman *et al.* [78], Slaton *et al.* [79], and Sims and Gamon [42], who found relatively weak correlations between NIR reflectance and the thickness of leaves' layers of other plant species, and also with the conclusions of Nobel *et al.* [80], Slaton *et al.* [79], Castro and Sanchez-Azofeifa [81], and Ollinger [82], which emphasized the importance of the correlation between NIR reflectance and the normalized area of the intercellular air spaces within the spongy mesophyll. The spectral significance of the age-dependent variability in intercellular void areas among leaves is manifested by its contribution on NIR reflectance. For example, in their study on spectroscopic monitoring of osmotic treatments, Gausman *et al.* [51] suggested that leaf maturation and internal structure changes have negatively affected their spectral representations. Similarly, in their study on predicting leaf water content through reflectance measurements, Thomas *et al.* [83] suggested that the poor correlation that was found was presumably the cause of leaf structural variations during development. Thus, overlooking the spectral differences between young and old leaves might prove to be a significant source of bias in reflectance measurements [41].

Growth regulation is also likely to affect many common vegetative spectral indices, especially those that are derived from NIR reflectance, absorbance, or transmittance data to monitor plant physiology. For instance, the NDVI and WI, which were established as indicative parameters for plant status and are widely used (e.g., [8], [39], [41], [57], [58], [84]–[87]), were found to be significantly affected by leaf age and, therefore, in high growth rates, might produce values that are unrepresentative for most leaves in the canopy (Table 2). The NDII, MSI, and SIPI, however, showed little to no sensitivity to aging implications and, thus, have a clear advantage as indicators under differential growth rates. The index sensitivity results are in accordance with those found in a similar, leaf age-directed study by Liu *et al.* [41], wherein the SIPI was also found to be the most insensitive to leaf ontogenesis, while the NDVI was found to be affected by it. The impact of within-foliage spectral variability was assessed in the current study by regressing values of g_s_ against values of NDVI – two variables that have been correlated in previous *Vitis vinifera* studies (e.g., [57], [87]). Calculating the weighted-average NDVI value of all leaves in the control plant's canopy has resulted in a g_s_ value of 0.050 mol H_2_O m^−2^ s^−1^, which was 0.242 mol H_2_O m^−2^ s^−1^ lower than the weighted-average NDVI prediction of only the representative leaves (around 45% of the canopy; Figure 5). Although a smaller difference in g_s_ was apparent in the case of the water deficit group, due to a higher ratio of representative leaves, the inclusion of all leaves in the canopy has resulted in a weighted-average NDVI that nearly zeroed the g_s_ of otherwise 0.057 mol H_2_O m^−2^ s^−1^. Similarly, when assessing potential physiological deviations in WI-based regressions of other studies, it seems that the inclusion of younger leaves in the predictions of Rodriguez-Pérez *et al.* [8] will result in a 30–40% higher water content as percent of total fresh mass (WCt), and their inclusion in the predictions of Sims and Gamon [84] will result in a 0.5 kg m^−2^ higher water content of thin tissues. These distinctions strongly imply that the use of spectral indices without considering the inherent variability within plants' canopies might lead to either an under- or over-estimation of health status parameters [41]. Furthermore, special attention should be given to inter-treatment comparative images taken by satellite or airborne remote sensors of very high spatial resolution, which are generally limited to the upper part of the foliage and suffer from background effects, complex canopy geometry issues, and disadvantageous viewing angles [49], [88], [89].

As was demonstrated in this study, growth regulation can break the age-position correlation across stress treatments, leading to incomparable physiological values. Although the implications of vegetative growth inhibition were discussed here in light of water deficit alone, the findings of this research are not limited to any other biotic or abiotic factor, as plant development can be affected by diseases [90], shoot trimming [91], pests [92], defoliation [93], [94], light deficiency [95], and many other stressing agents. Moreover, since growth rate differences may occur between various cultivars of the same species (e.g., [96], [97]), and since spectral studies may include combinations of deciduous and non-deciduous plants, growth regulation becomes an even more common and complex problem altogether. Ergo, before attempting to compare leaves of different plants and treatments, future high spatial resolution-remote sensing works should take into account the specific spectral variability within the canopies of their choice, with respect to the specific type of stress induced. This idea may prove to be more difficult to implement than the protocol of the current study, as age- and stress-induced changes in leaf structure, pigment concentrations, and biochemical components need to be spectrally separated. However, combining high spectral resolution spectroscopy with advanced radiative models (e.g., [98], [99]) or advanced statistical tools [5] may lead to a successful discrimination between the impacts of maturation and stress, thus allowing a better understanding of differential growth rates' spectral effects.

## Summary and Conclusions

In this study, the spectral-physiological implications of overlooking differential growth rates across stress treatments were discussed. As was shown, the variance in the growth rates has gradually established a new and distinct correlation between leaf age and position for each of the treatments, depicted by NIR reflectance differences among leaves of the same nodal degree. The spectral difference between young leaves of both treatment groups was anatomically attributed to the difference between their spongy mesophylls' intercellular void areas, which was affected by leaf maturation and controlled NIR scattering. Differences in the sensitivity level of derived reflectance indices to the age-position effect have demonstrated that some of them may have more potential than others under terms of differential growth rates, and are likely to predict less biased physiological values, especially for annual and deciduous species. Therefore, the methodological flaw that was described in this study – manifested by many forms of stress on the one hand and rarely discussed in the remote sensing literature on the other hand – should be taken into account in future precision agriculture studies.
